# UnetTransCNN: integrating transformers with convolutional neural networks for enhanced medical image segmentation

**DOI:** 10.3389/fonc.2025.1467672

**Published:** 2025-07-10

**Authors:** Yi-Hang Xie, Bo-Song Huang, Fan Li

**Affiliations:** ^1^ School of Mathematics,Statistics and Mechanics, Beijing University of Technology, Beijing, China; ^2^ School of Electronic and Information Engineering, Beijing Jiaotong University, Beijing, China; ^3^ School of Computer Science, Inner Mongolia University, Hohhot, China; ^4^ College of Software, Inner Mongolia University, Hohhot, China

**Keywords:** fully convolutional neural networks, transformer, medical image segmentation, 3D image, feature fusion

## Abstract

**Introduction:**

Accurate segmentation of 3D medical images is crucial for clinical diagnosis and treatment planning. Traditional CNN-based methods effectively capture local features but struggle with modeling global contextual dependencies. Recently, transformer-based models have shown promise in capturing long-range information; however, their integration with CNNs remains suboptimal in many hybrid approaches.

**Methods:**

We propose UnetTransCNN, a novel parallel architecture that combines the strengths of Vision Transformers (ViT) and Convolutional Neural Networks (CNNs). The model features an Adaptive Fourier Neural Operator (AFNO)-based transformer encoder for global feature extraction and a CNN decoder for local detail restoration. Multi-scale skip connections and adaptive global-local coupling units are incorporated to facilitate effective feature fusion across resolutions. Experiments were conducted on the BTCV and MSD public datasets for multi-organ and tumor segmentation.

**Results:**

UnetTransCNN achieves state-of-the-art performance with an average Dice score of 85.3%, outperforming existing CNN- and transformer-based models on both large and small organ structures. The model notably improves segmentation accuracy for challenging regions, achieving Dice score gains of 6.382% and 6.772% for the gallbladder and adrenal glands, respectively. Robustness was demonstrated across various hyperparameter settings and imaging modalities.

**Discussion:**

These results demonstrate that UnetTransCNN effectively balances local precision and global context, yielding superior segmentation performance in complex anatomical scenarios. Its parallel design and frequency-aware encoding contribute to enhanced generalizability, making it a promising tool for high-precision medical image analysis.

## 1 Introduction

With the rapid advancements in the fields of computer science and medical imaging, medical imaging technologies such as computed tomography (CT) Vaninsky (1) and magnetic resonance imaging (MRI) Khuntia et al. (2) have emerged as indispensable tools in medical research Lim and Zohren (3), clinical diagnosis Masini et al. (4), and surgical planning Torres et al. (5). These technologies allow non-invasive imaging of internal tissues and organs’ physiological states, representing a key advance in merging computer science with medicine Zeng et al. (6), Shen et al. (7).

The emerging technologies Challu et al. (8), Azad et al. (9) concurrently introducing new challenges such as the need for classification and processing of diagnostic results. Image classification techniques play a pivotal role in autonomously comprehending the content of images to a certain extent. They enable effective identification of pathological regions within medical images, thereby assisting physicians in efficient diagnosis Stankeviciute et al. (10). However, the reality of medical imaging encompasses a diverse array of image types Wu et al. (11), often requiring the application of distinct processing and analytical approaches to differentiate between categories of medical images.

In recent years, advances in deep learning have renewed interest in medical image segmentation, drawing significant attention from researchers Wu et al. (12). Deep learning excels at automatically extracting features from complex data during training, leveraging multi-layered neural networks to create high-dimensional feature representations that boost segmentation performance Le Guen and Thome (13). This capability underpins deep learning-based medical image classification and grading, which supports diagnosis, speeds up image analysis, reduces patient wait times, and eases radiologists’ workloads.

We define key terms here: ‘CNN-based models’ refer to architectures relying on Convolutional Neural Networks (CNNs) for feature extraction, emphasizing local patterns, while ‘Transformer-based models’ use Transformer architectures to capture global contextual relationships via self-attention mechanisms. These definitions will be applied consistently throughout this manuscript.

In practical medical image segmentation, precise classification demands both local lesion details and global contextual information—a challenge for standard CNN-based models. Although CNNs excel at local feature extraction, their inductive bias limits their ability to capture global dependencies, hindering further performance gains. Inspired by the success of Transformer-based models like ViT Stankeviciute et al. (10) in natural image tasks, recent studies have integrated these with CNN-based approaches for medical imaging, often matching or exceeding CNN performance. For instance, TransUNet Du et al. (14), the first to combine Transformer-based and CNN-based strengths [via U-Net Fan et al. (15)], embeds a Transformer in the encoder. Similarly, MCTransformer Elsworth and Güttel (16) unfolds CNN-extracted multiscale features into tokens for Transformer processing.

Despite these advances, integrating local and global features remains challenging when CNNs and Transformers are simply concatenated or embedded. To overcome this, we propose UnetTransCNN, a novel parallel architecture that simultaneously extracts local features (via a CNN-based module) and global features (via a Transformer-based module). Unlike prior models such as TransUNet or MCTransformer, which fuse sequentially, our design optimizes CNNs for local detail and Transformers for global context in parallel. We further introduce adaptive global-local coupling units to dynamically fuse features from both pathways across multiple scales. This enhances accuracy in segmenting complex structures and improves generalizability across diverse medical imaging tasks. The contributions of this paper can be summarized as follows:

### 1.1 Proposed UnetTransCNN model

We propose the novel UnetTransCNN model that utilizes CNN and ViT (Vision Transformer) in parallel to extract both local and global features from medical images. This dual-path approach ensures a comprehensive feature analysis, enhancing the segmentation accuracy.

### 1.2 Application to 3D medical image segmentation

We specifically adapt the UnetTransCNN model for 3D medical image segmentation. In order to fit the unique structure of 3D volumes, we incorporate specialized adaptations such as volumetric convolutions and 3D positional encodings, significantly improving the model’s effectiveness in handling spatial relationships within medical volumes.

### 1.3 Design and implementation of experiments

We design a variety of experiments to demonstrate the superiority of our model. Our UnetTransCNN achieves superior metrics on two public datasets, the BTCV and MSD. Additionally, it demonstrates excellent robustness across various hyperparameters when compared to existing popular models, thereby proving its efficacy in real-world medical applications.

## 2 Related work

### 2.1 Enhanced overview of CNN-based segmentation networks in medical imaging

Since the inception of the seminal U-Net architecture, the realm of medical imaging has witnessed profound advancements through the adoption of Convolutional Neural Network (CNN)-based techniques for segmenting 2D and 3D images, as documented in numerous studies Wu et al. (11), Rahman et al. (17). In addressing the intricacies of volume-level segmentation, the innovative 2.5D approach has been introduced. This method ingeniously integrates three distinct perspectives of each voxel via a tri-planar architecture, offering a nuanced view beyond conventional methods. Meanwhile, 3D segmentation strategies Ding et al. (18) directly engage with volumetric images, harnessing a compendium of 2D slices or imaging modalities to achieve a comprehensive analysis.

To adeptly navigate the challenges of downsampling within images, the research community has ventured into the expansion of dimensional concepts, embracing multi-channel and multi-path models. This evolution signifies a stride towards capturing a richer tapestry of image features. Furthermore, the quest for effectively leveraging 3D contextual insights, while judiciously managing computational resources, has propelled the exploration of hierarchical structures. Innovative methodologies have surfaced, incorporating tactics like multi-scale feature extraction and the synergistic amalgamation of diverse frameworks. For example, reference Wu and Xu (19) highlights a pioneering multi-scale framework adept at discerning information across various resolutions, specifically tailored for pancreas segmentation.

These cutting-edge approaches mark a significant milestone in the field of 3D medical image segmentation. They ambitiously aim to navigate the complexities associated with spatial context and the challenges posed by low-resolution imagery, paving the way for groundbreaking research endeavors in multi-level 3D medical image analysis.

Despite the notable success achieved by these methods, they still suffer from a limitation in learning global context and long-range spatial dependencies. This issue can significantly impact the segmentation performance for challenging tasks. Therefore, to further improve segmentation performance Wu et al. (12), researchers are actively exploring new methods and techniques to effectively capture global contextual information and long-range spatial dependencies, thereby enhancing the accuracy and robustness of medical image segmentation.

### 2.2 Vision transformers

In recent years, visual Transformer models have attracted widespread attention and research in the computer vision field. Dosovitskiy et al. demonstrated excellent performance in image classification tasks by pretraining and fine-tuning a pure Transformer model Lara-Benítez et al. (20). Furthermore, Transformer-based end-to-end object detection models have shown significant advantages in multiple benchmark tests Cirstea et al. (21). To further improve performance, researchers have proposed a series of hierarchical visual Transformer models that gradually reduce the feature resolution in Transformer layers and employ subsampling attention modules to achieve this Fei et al. (22). However, unlike these methods, the representation size in the UnetTransCNN encoder remains unchanged across all Transformer layers. In Section 3, we introduce a method that uses deconvolution and convolution operations to change the feature resolution.

In the realm of image analysis, Transformer-based models have gone beyond image classification and object detection to make significant strides in 2D image segmentation. The SETR model, introduced by Wu et al. (23), leverages a pretrained Transformer encoder alongside a CNN-based decoder variant for semantic segmentation. Meanwhile, Du et al. (14) has pioneered a multi-organ segmentation technique by integrating a Transformer layer within the U-Net architecture’s bottleneck section Kurle et al. (24). Additionally, Xu et al. (25) has developed a strategy that distinguishes the roles of CNN and Transformer, merging their outcomes Wu et al. (26). Godunov and Bohachevsky (27) has innovated an axial attention mechanism rooted in Transformers for 2D medical image segmentation.

Our model sets itself apart from these approaches in crucial ways: (1) UnetTransCNN is tailor-made for 3D segmentation, directly handling volumetric data; (2) It positions the Transformer as the main encoder within the segmentation framework, linking it to the decoder with skip connections rather than merely as an attention component; (3) UnetTransCNN bypasses the need for a backbone CNN for input sequence creation, opting instead for direct use of tokenized patches.

Focusing on 3D medical image segmentation, Cirstea et al. (21) introduced a framework that utilizes a backbone CNN for initial feature extraction, then processes the encoded representation through a Transformer, concluding with a CNN decoder for segmentation prediction Moin and Mahesh (28). In a similar vein, Khan et al. (29) has developed a technique for the semantic segmentation of brain tumors, employing a Transformer within the bottleneck phase of a 3D encoder-decoder CNN model Rogallo and Moin (30). Differing from these methodologies, our approach forges a direct link between the Transformer’s encoding representation and the decoder via skip connections. This strategic decision empowers our model to fully harness the Transformer’s representational capabilities, driving superior performance in 3D medical image segmentation tasks.

## 3 Method

**Figure 1:**
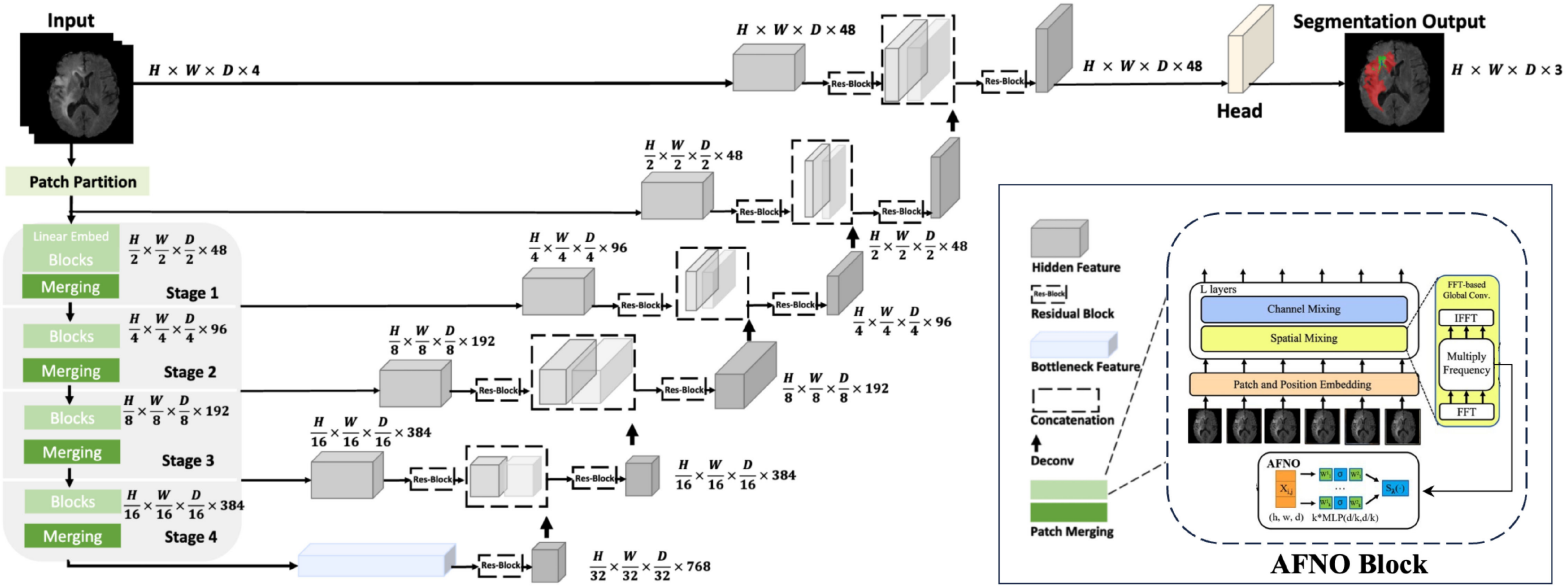
Overview of the UnetTransCNN architecture. The input to our model is 3D multi-modal MRI images with 4 channels. The UnetTransCNN creates non-overlapping patches of the input data and uses a patch partition layer to create windows of a desired size for computing Fourier-based attention in the AFNO encoder. The encoded feature representations in the AFNO are fed to a CNN-decoder via skip connections at multiple resolutions.

Our proposed model, named UnetTransCNN, employs an innovative approach that combines the global context capture capability of Transformer with the powerful local feature extraction capability of CNN, aiming to improve the accuracy and efficiency of medical image segmentation. The details of our model are demonstrated in **Figure 1**.

### 3.1 Encoder architecture

Integrating the Adaptive Fourier Neural Operator (AFNO) into the encoder enhances its ability to process 3D medical imagery using spatial and frequency domain information. The process begins by dividing the input image into non-overlapping cubic patches of size *P* × *P* × *P*, which are transformed into K-dimensional embedding vectors via:


(1) $$E_{\text{patch}}=\text{Flatten}\left(x_{v}\right)\cdot W_{\text{proj}}+E_{\text{pos}}$$

Here, *x_v_* represents the cubic patches from the input, *W*_proj_ is the projection matrix mapping patch data to the embedding space, and *E*_pos_ encodes the spatial positions of the patches. This process is mathematically defined in Equation (1).

These embeddings are then processed through Transformer layers, each with a multi-head self-attention (MSA) mechanism and a multi-layer perceptron (MLP), strengthening the model’s understanding of global dependencies. The operations in each Transformer layer are given by: These steps are formally described in Equations (2) and (3).


(2) $$z_{i}^{'}=\text{MSA}\left(\text{Norm}\left(z_{i-1}\right)\right)+z_{i-1}$$

(3) $$z_{i}=\text{MLP}\left(\text{Norm}\left(z_{i}^{'}\right)\right)+z_{i}^{'}$$

where Norm stands for the layer normalization process, and *i* represents the index of the Transformer layer in sequence.

To integrate the complex Fourier formula and AFNO’s adaptive processing, the embeddings undergo a Fourier transform after the initial MLP transformation and before the Transformer layers. This enables the encoder to adaptively handle spatial frequencies, performed as follows:

1. Discrete Fourier Transform (DFT) of the embedding vector to shift the representation from the spatial to the frequency domain see Equation (4):


(4) $$F\left(k\right)=\sum_{n=0}^{N-1}e\left(n\right)\cdot e^{-\frac{2\pi i}{N}nk}$$

2. Adaptive Modulation in the frequency domain, applying learned weights to each frequency component to emphasize relevant spatial frequencies see Equation (5):


(5) $$F_{\text{mod}}\left(k\right)=F\left(k\right)\cdot W\left(k\right)$$

3. Inverse DFT (IDFT) to convert the modulated frequency components back to the spatial domain, generating enhanced embeddings see Equation (6)


(6) $$e^{\prime }\left(n\right)=\frac{1}{N}\sum_{k=0}^{N-1}F_{\text{mod}}\left(k\right)\cdot e^{\frac{2\pi i}{N}nk}$$

The UnetTransCNN model balances global patterns and local details by manipulating data in both frequency and spatial domains, critical for precise medical image segmentation where macroscopic and microscopic features must be accurately captured.

The encoding process relies on the Discrete Fourier Transform (DFT) and Inverse Discrete Fourier Transform (IDFT). The DFT shifts image analysis to the frequency domain, revealing global patterns like periodic textures and edges not easily seen in the spatial domain. This allows the encoder to effectively modulate these broad features. The IDFT then converts the adjusted frequency data back to the spatial domain, preserving the image structure while embedding enhanced features—essential for segmentation, as without it, frequency-domain improvements wouldn’t translate to spatial results.

Through this process, the AFNO-transformer optimizes the encoder to leverage both local and global information, improving its ability to handle complex spatial relationships in volumetric medical data. This Fourier transform integration drives the UnetTransCNN model’s superior performance in medical image segmentation.

### 3.2 Decoder architecture

The decoder uses Convolutional Neural Networks (CNNs) to extract and restore local image features for precise segmentation. It operates through decoding stages that fuse features from the corresponding encoder stage (via skip connections) with outputs from the previous decoding stage. This process is defined by see Equation (7):


(7) $$F_{\text{dec}}^{i}=\text{Conv}\left(\text{Up}\left(F_{\text{dec}}^{i-1}\right)⊕F_{\text{enc}}^{i}\right),$$

where $F_{\text{dec}}^{i}$ is the feature map at the decoder’s *i*th layer, Conv refines the feature maps, Up upsamples to increase resolution, ⊕ merges features, and $F_{\text{enc}}^{i}$ is the encoder’s *i*th layer feature map linked by skip connections.

After progressing through these stages, a final 1×1×1 convolution layer processes the output to predict semantic labels for each voxel, converting feature maps into class probabilities (see Equation (8)):


(8) $$Y_{\text{pred}}=\text{Softmax}\left({\text{Conv}}_{1\times 1\times 1}\left(F_{\text{dec}}^{\text{final}}\right)\right),$$

Here, *Y*_pred_ represents the voxel-wise predictions, and Softmax normalizes the final convolution’s logits into a probability distribution across classes, ensuring accurate segmentation of medical images.

### 3.3 Model application overview

The UnetTransCNN-CNN architecture adeptly integrates the distinct advantages of Transformers and Convolutional Neural Networks (CNNs), harnessing Transformers for their superior global contextual understanding and utilizing CNNs for their acute precision in local detail processing. This dual-approach is particularly advantageous for medical imaging tasks, where it adeptly manages the intrinsic complexity and variability of medical image structures. This results in enhanced segmentation accuracy and improved model reliability. Further, the meticulous development of our model is underpinned by robust mathematical formulations and comprehensive process elucidations, as delineated in prior sections. Consequently, UnetTransCNN-CNN emerges as a profoundly efficient and precise methodology for tackling medical image segmentation challenges, particularly effective in scenarios involving complex anatomical structures. The operational dynamics of the model are succinctly encapsulated in **Algorithm 1**, providing a clear workflow that underscores the model’s computational strategy.

Algorithm 1 **UnetTransCNN for Medical Image Segmentation with AFNO.**

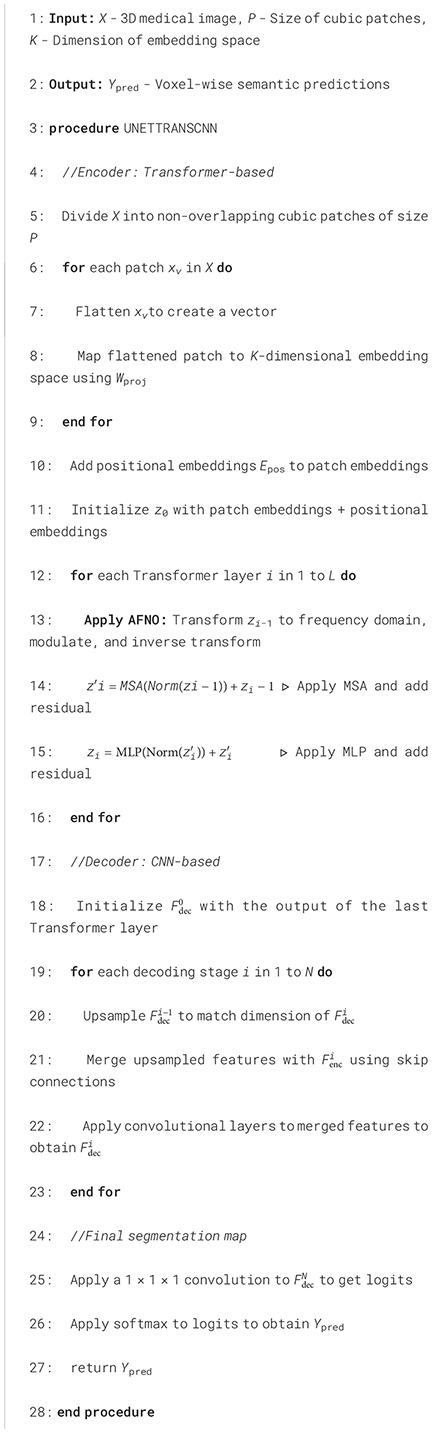

### 3.4 Model Workflow Example

**Input:** The input to the model is a 3D multi-modal MRI image with dimensions *H* ×*W* ×*D* ×*C*, where *C* = 4 represents the different imaging modalities (e.g., T1, T2, FLAIR). For example, an input could have dimensions 128 × 128 × 128 × 4.

**Patch Partition** The input data is divided into non-overlapping patches of size 4 × 4 × 4, each patch serving as a token for subsequent processing. The resulting patch dimensions are projected into a feature space through a linear embedding.

**AFNO Encoder** The encoded features pass through the AFNO encoder, which consists of four hierarchical stages:

- **Stage 1:** Produces feature maps with dimensions *H/*2 × *W/*2 × *D/*2 × 48. This stage applies Fourier-based global convolution and spatial mixing using the AFNO block.
- **Stage 2:** Downsamples the spatial resolution to *H/*4 × *W/*4 × *D/*4 × 96 while increasing feature depth.
- **Stage 3:** Further reduces spatial dimensions to *H/*8 × *W/*8 × *D/*8 × 192.
- **Stage 4:** Final encoding stage with feature dimensions *H/*16 × *W/*16 × *D/*16 × 384.

Each stage uses patch merging for downsampling and captures multi-scale representations through Fourier domain operations.

**CNN Decoder** The decoder progressively upsamples the feature maps to the original spatial resolution. Each upsampling stage incorporates skip connections from the corresponding encoder stage, ensuring that both local and global information are retained:

- **Stage 1 Decoder:** Receives encoder outputs with dimensions *H/*16 × *W/*16 × *D/*16, upsampled and concatenated with encoder outputs from Stage 3.
- **Stage 2 Decoder:** Further upsamples to *H/*4 × *W/*4 × *D/*4, integrating features from Stage 2.
- **Stage 3 Decoder:** Restores dimensions to *H/*2 × *W/*2 × *D/*2, using features from Stage 1.

### 3.5 Comparison with previous hybrid approaches

The integration of CNN-based and Transformer-based models has been explored in prior works like TransUNet Du et al. (14), which combines a Transformer with a U-Net architecture to leverage both local and global features for medical image segmentation. While TransUNet demonstrates notable success, it has limitations that hinder its performance in certain scenarios. Specifically, its heavy reliance on Transformer layers prioritizes global contextual information, often at the expense of fine-grained local details. This imbalance can lead to suboptimal segmentation of intricate structures where precise localization is critical, as the CNN component in TransUNet is not sufficiently optimized to compensate for the Transformer’s focus on broader patterns.

In contrast, UnetTransCNN addresses these shortcomings through a more balanced and refined design. Our approach enhances local feature extraction by incorporating a strengthened CNN-based backbone, tailored to capture detailed spatial information effectively. Simultaneously, we optimize the Transformer-based module to align global contextual understanding with the spatial hierarchies inherent in medical images. This dual-pathway architecture, supported by adaptive global-local coupling units, ensures a complementary integration of local and global features. Unlike TransUNet’s sequential fusion, UnetTransCNN processes these features in parallel, allowing for a more precise and context-aware segmentation. These improvements enable UnetTransCNN to outperform previous hybrid approaches, particularly in tasks requiring both detailed localization and comprehensive contextual awareness.

## 4 Experiments

### 4.1 Dataset

**Figure 2:**
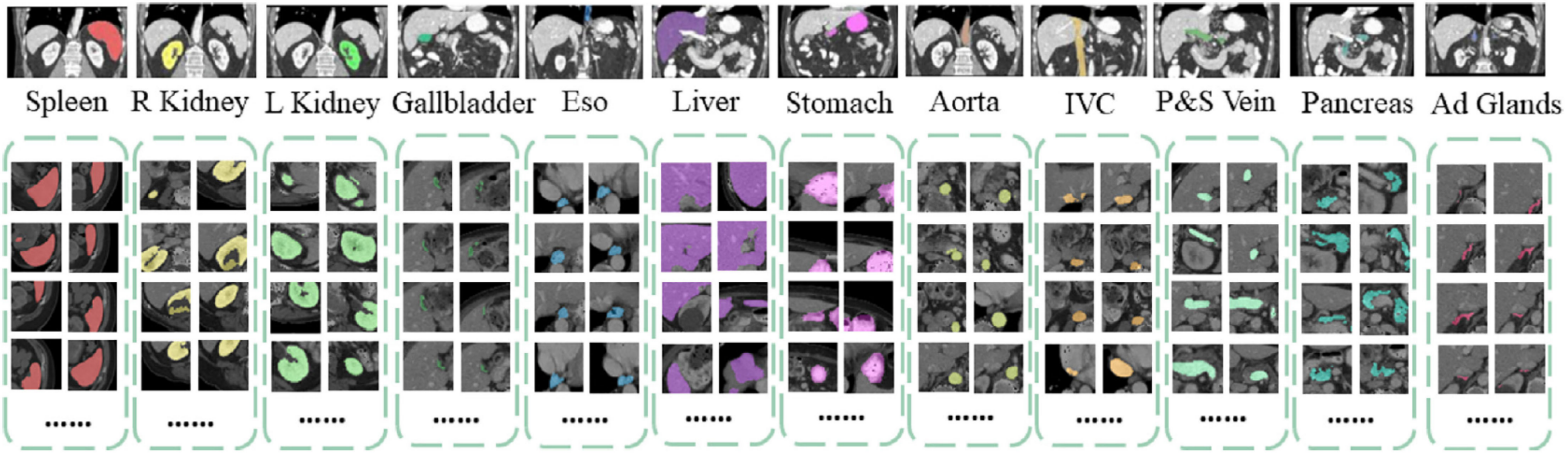
Dataset visualization of segmentation.

**Figure 2** depicts a high-dimensional medical computed tomography (CT) image dataset, specifically designed for the segmentation of major abdominal organs for medical image analysis, originating from the Abdominal Organ Segmentation Challenge (BTCV) van der Hoef et al. (31). The dataset encompasses multiple abdominal organs, including the spleen, right kidney (R Kidney), left kidney (L Kidney), gallbladder, esophagus (Eso), liver, stomach, aorta, inferior vena cava (IVC), portal and spleen vein (P&S Vein), pancreas, and adrenal glands (Ad Glands).

Each set of images displays multiple consecutive CT slices from the same subject, with each organ marked in a specific color for differentiation. These color-coded markings allow researchers to quickly identify and analyze the boundaries and morphology of the organs. For instance, the spleen is marked in red, kidneys in yellow, and the liver in purple, with each color chosen to optimize visual contrast for algorithmic processing.

The dimensions of this dataset can be described in several aspects:

1. Spatial dimension: The images of each organ consist of a series of cross-sections arranged along the body’s vertical axis, showcasing the three-dimensional structure of the organs.
2. Time/sequence dimension: Although not directly shown in this image, in practice, such datasets may include temporal sequence information, representing dynamic scans over time.
3. Grayscale/intensity dimension: CT images present different grayscale intensities based on the varying degrees of X-ray absorption by tissues, reflecting differences in tissue density.
4. Annotation dimension: The CT images of each organ in the dataset come with detailed manual annotations providing ground truth information for training and validating automatic image segmentation algorithms.
5. Patient/sample dimension: The dataset includes scans from multiple patients, enhancing sample diversity and aiding algorithms in better generalizing to unseen samples.

The MSD dataset, referenced in Gao and Ma (32), is a critical resource for the brain tumor segmentation task, encompassing a wide array of multi-modal, multi-site MRI and CT data. This dataset is specifically curated with 484 MRI scans, each offering a variety of modalities including FLAIR, T1-weighted (T1w), T1-weighted post-contrast (T1gd), and T2-weighted (T2w) images, accompanied by detailed ground truth labels. These labels facilitate the segmentation of glioma, delineating areas of necrotic/active tumor and edema regions. The MRI images within this dataset are characterized by a uniform voxel spacing of 1.0 × 1.0 × 1.0 mm^3^, ensuring consistency and precision in volumetric analysis Kim et al. (33), Wu et al. (34), Silva (35). In preparation for training, the dataset undergoes a standard pre-processing step where voxel intensities are normalized using the z-score method. This meticulous preparation allows the segmentation task to be framed as a 3-class challenge, incorporating a 4-channel input to effectively differentiate between the various tumor regions and healthy brain tissue.

To further evaluate the generalization capability of the model, we also use the KiTS19 (36) dataset Yang and Farsiu (37). This dataset is widely used for medical image segmentation tasks and includes a diverse range of kidney tumor cases, which can help evaluate the model’s performance on complex anatomical structures. KiTS19 contains 210 contrast-enhanced CT scans of patients with kidney tumors. The dataset includes annotations for kidney and tumor regions, making it suitable for evaluating segmentation models. The diversity in tumor sizes, shapes, and locations provides a robust test for the generalization capability of the model.

### 4.2 Evaluation metrics

In our research, we meticulously assess the accuracy of segmentation results by employing the Dice coefficient and the 95% Hausdorff Distance (HD), as delineated in Zeng et al. (6). The Dice coefficient is utilized to quantitatively evaluate the similarity between the actual (ground truth) and predicted segmentation maps, defined for voxel *i* as *T_i_* for the actual values and *S_i_* for the predicted values, respectively. The formula for the Dice coefficient is given as follows (see Equation (9)):


(9) $$\text{Dice}\left(T,S\right)=\frac{2\sum_{i=1}^{I}T_{i}S_{i}}{\sum_{i=1}^{I}T_{i}+\sum_{i=1}^{I}S_{i}},$$

where *I* is the total number of voxels. This coefficient ranges from 0 to 1, where a value of 1 indicates perfect overlap between the actual and predicted segmentation, and a value of 0 indicates no overlap.

The 95% Hausdorff Distance (HD) measures the spatial distance between the surface points of the actual and predicted segmentation, offering a robust metric for the maximum discrepancy between these two point sets. It is defined as (see Equation (10)):


(10) $$\text{HD}\left(T^{\prime },S^{\prime }\right)=\text{max }\left(\underset{t^{\prime }\in T^{\prime }}{\max}\underset{s^{\prime }\in S^{\prime }}{\min}|t^{\prime }-s^{\prime }\left|,\underset{s^{\prime }\in S^{\prime }}{\max}\underset{t^{\prime }\in T^{\prime }}{\min}|s^{\prime }-t^{\prime }\right|\right),$$

where *T* ^′^ and *S* ^′^ represent the sets of actual and predicted surface points, respectively. The HD is particularly sensitive to outliers; therefore, by calculating the 95th percentile of these distances, we mitigate the influence of extreme values, leading to a more representative measurement of model performance. This adjusted metric, focusing on the 95th percentile, effectively reduces the impact of anomalies, providing a more robust and reliable evaluation of the segmentation precision.

### 4.3 Implementation details

Our UnetTransCNN model was implemented on a high-performance computing cluster equipped with NVIDIA A100 Tensor Core GPUs, each boasting 40 GB of memory, which is particularly crucial for processing large 3D medical images and complex models. We utilized PyTorch as the deep learning framework, opting for an input block size of 64 × 64 × 64 voxels and an embedding dimension of 768, along with 12 transformer layers to capture complex patterns and dependencies. The model underwent training on two benchmark datasets: the Multi Atlas Labeling Beyond The Cranial Vault (BTCV) and the Medical Segmentation Decathlon (MSD). For both datasets, we partitioned the data into training and testing sets, using 80% of the data for training and the remaining 20% for testing. This split was carefully chosen to ensure that the model was evaluated on a diverse range of images that were not seen during the training phase, thus reflecting a realistic assessment of the model’s performance on unseen data. Additionally, diverse 3D medical images from these datasets are used for multi-organ and tumor segmentation tasks. To enhance the model’s robustness and prevent overfitting, we also applied data augmentation techniques such as random rotations, scaling, and elastic deformations. Throughout the training process, we employed the AdamW optimizer with a learning rate of 1*e* − 4 and a weight decay of 0.01, using an early stopping strategy to prevent overfitting across 150 training epochs. This detailed implementation strategy ensured the effective training and evaluation of the model, leveraging the computational power of NVIDIA A100 GPUs to meet the challenges of 3D medical image segmentation.

For the compared baselines, we adhered to the official configurations and hyperparameters provided in the original papers or publicly available repositories of the competing methods. We ensured uniform dataset splits (80% training and 20% validation) across all methods to eliminate variability introduced by differing data partitions. Further, all methods were evaluated using the Dice coefficient and Hausdorff distance (95%), ensuring consistent and comparable performance assessments. To ensure fairness and consistency across all experiments, we trained all methods on all datasets for 600 epochs.

### 4.4 Main results

In the rigorous evaluation conducted during the Standard Competition, our novel UnetTransCNN model has set a benchmark, emerging as the frontrunner by achieving an unparalleled average Dice score of 85.3% across various organs. This achievement underscores the model’s exceptional capability in handling the complexities of medical image segmentation. Specifically, UnetTransCNN has displayed a noteworthy advantage in segmenting larger organs. A quantitative summary of these results is presented in **Table 1**. For instance, it outshines the second-best baselines with significant margins in the segmentation of the spleen, liver, and stomach, registering improvements in the Dice score by 1.043%, 0.830%, and 2.125%, respectively. These figures not only attest to the model’s precision but also its robustness in accurately identifying and delineating the contours of larger organ structures.

**Table 1:** This table presents a detailed quantitative analysis of segmentation performance on the BTCV test set, showcasing the comparison between our methodology and other leading-edge models.

Methods	Spl	RKid	LKid	Gall	Eso	Liv	Sto	Aor	IVC	Veins	Pan	AG	Avg.
SETR NUP Sahoo et al. (38)	0.931	0.890	0.897	0.652	0.760	0.952	0.809	0.867	0.745	0.717	0.719	0.620	0.796
SETR PUP Xu et al. (39)	0.929	0.893	0.892	0.649	0.764	0.954	0.822	0.869	0.742	0.715	0.714	0.618	0.797
SETR MLA Hajirahimi and Khashei (40)	0.930	0.889	0.894	0.650	0.762	0.953	0.819	0.872	0.739	0.720	0.716	0.614	0.796
nnUNet Godahewa et al. (41)	0.942	0.894	0.910	0.704	0.723	0.948	0.824	0.877	0.782	0.720	0.680	0.616	0.802
ASPP Zhou et al. (42)	0.935	0.892	0.914	0.689	0.760	0.953	0.812	0.918	0.807	0.695	0.720	0.629	0.811
TransUNet Sirisha et al. (43)	0.952	**0.927**	0.929	0.662	0.757	0.969	0.889	0.920	0.833	0.791	0.775	0.637	0.838
CoTr w/o CNN encoder Khan et al. (29)	0.941	0.894	0.909	0.705	0.723	0.948	0.815	0.876	0.784	0.723	0.671	0.623	0.801
CoTr* Khan et al. (29)	0.943	0.924	0.929	0.687	0.762	0.962	0.894	0.914	0.838	**0.796**	**0.783**	0.647	0.841
CoTr Khan et al. (29)	0.958	0.921	0.936	0.700	0.764	0.963	0.854	**0.920**	0.838	0.787	0.775	0.694	0.844
**UnetTransCNN**	**0.968**	0.924	**0.941**	**0.750**	**0.766**	**0.971**	**0.913**	0.890	**0.847**	0.788	0.767	**0.741**	**0.856**
RandomPatch Li et al. (44)	0.963	0.912	0.921	0.749	0.760	0.962	0.870	0.889	0.846	0.786	0.762	0.712	0.844
PaNN Cao et al. (45)	0.966	0.927	0.952	0.732	0.791	0.973	0.891	0.914	0.850	0.805	0.802	0.652	0.854
nnUNet-v2 Eldele et al. (46)	0.972	0.924	**0.958**	0.780	0.841	0.976	0.922	0.921	0.872	0.831	0.842	0.775	0.884
nnUNet-dys3 Eldele et al. (46)	0.967	0.924	0.957	0.814	0.832	0.975	0.925	0.928	0.870	0.832	**0.849**	0.784	0.888
DconnNet Yang and Farsiu (37)	0.968	0.931	0.952	0.818	0.856	0.977	0.918	0.934	0.882	0.843	0.803	0.795	0.875
**UnetTransCNN**	**0.972**	**0.942**	0.954	**0.825**	**0.864**	**0.983**	**0.945**	**0.948**	**0.890**	**0.858**	0.799	**0.812**	**0.891**

The evaluation focuses on the benchmarks established for both the Standard and Free Competitions, situating our approach in the context of these predefined standards. It’s imperative to highlight that the foundation for all comparisons involving SETR models was the ViT-B-16 architecture. A pivotal aspect of this analysis involves the segmentation results across a diverse array of organs including the spleen, right and left kidneys (RKid and LKid), gallbladder (Gall), esophagus (Eso), liver (Liv), stomach (Sto), aorta (Aor), inferior vena cava (IVC), the collective veins (encompassing portal and splenic veins), pancreas (Pan), and the adrenal gland (AG). These results were meticulously compiled from the BTCV leaderboard, ensuring a comprehensive and accurate benchmarking against the current state-of-the-art models.

Bold values indicate the best performance among all compared methods in each category.

**Figure 3:**
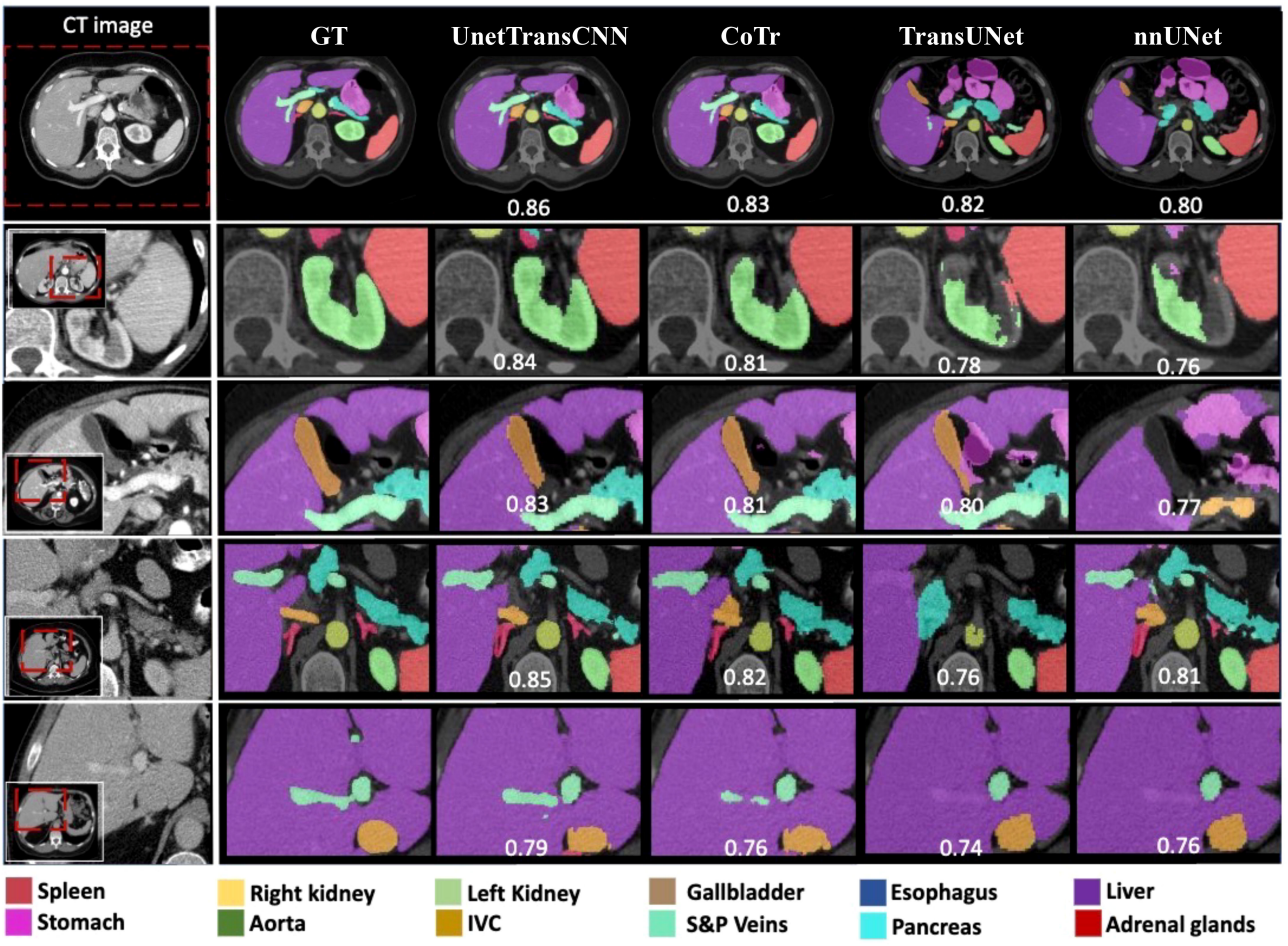
This image compares organ segmentation in CT scans across various deep learning models. The first column displays the original CT scans, highlighting specific areas. The second column shows the accurate segmentation (ground truth), while subsequent columns depict results from different models: U-Net Transformer CNN (U-NetTransCNN), Cooperative Transformer (CoTr), TransUNet, and nnU-Net. Predictions are color-coded for different organs, listed at the bottom. Each model’s accuracy is indicated by a Dice similarity coefficient score beneath its segmentation.

Detailed segmentation results are illustrated in **Figures 2**, **3**. Furthermore, UnetTransCNN’s proficiency extends to the segmentation of smaller organs, where it remarkably surpasses the second-best baselines by considerable margins of 6.382% and 6.772% in the Dice score for the gallbladder and adrenal glands, respectively. Such impressive performance metrics highlight the model’s detailed attention to the finer aspects of medical imaging, ensuring that even the smallest organs are segmented with high accuracy. These outcomes collectively reinforce the superior segmentation capability of UnetTransCNN, marking a significant advancement in the field of medical image analysis by delivering precise and reliable organ delineation.

In the Standard Competition, we conducted a comprehensive performance analysis of UnetTransCNN in comparison to CNN and transformer-based baselines. Impressively, UnetTransCNN establishes a new state-of-the-art performance, achieving an average Dice score of 85.3% across all organs. Notably, our method demonstrates remarkable superiority in segmenting large organs, such as the spleen, liver, and stomach, surpassing the second-best baselines by margins of 1.043%, 0.830%, and 2.125%, respectively, in terms of Dice score. Moreover, our method exhibits outstanding segmentation capability for small organs, outperforming the second-best baselines by impressive margins of 6.382% and 6.772% on the gallbladder and adrenal glands, respectively, in terms of Dice score. These results further highlight the exceptional performance of UnetTransCNN in accurately delineating organ boundaries. **Table 2** presents a full summary of segmentation scores across all organs in the BTCV dataset.

**Table 2:** Inference Speed Comparison on MSD Dataset.

Method	Inference Time (ms)	Speedup (%)
nnUNet	1620	–
TransUNet	1405	13.3%
CoTr	1202	25.8%
DconnNet	1100	32.1%
**UnetTransCNN (Ours)**	**987**	**39.1%**

Bold values indicate the best performance among all compared methods in each category.

In **Table 3**, we present a comparative analysis of UnetTransCNN, CNN, and transformer-based methodologies for brain tumor and spleen segmentation tasks using the MSD dataset. UnetTransCNN demonstrates superior performance compared to the closest baseline by an average margin of 1.5% across all semantic classes in brain segmentation. Detailed comparisons for brain tumor segmentation are reported in **Table 4**. Notably, UnetTransCNN exhibits exceptional accuracy in segmenting the tumor core (TC) subregion. Similarly, in spleen segmentation, UnetTransCNN surpasses the best competing methodology by at least 1.0% in terms of Dice score, indicating its superior segmentation capabilities. These results highlight the significant advancements achieved by UnetTransCNN in accurately delineating brain tumors and spleen regions.

**Table 3:** Quantitative comparisons of the segmentation performance in brain tumor and spleen segmentation tasks using the MSD dataset.

Task/Modality	Spleen Segmentation (CT)		Brain tumor Segmentation (MRI)							
Anatomy	Spleen		WT		ET		TC		ALL	
Metrics	Dice	HD95	Dice	HD95	Dice	HD95	Dice	HD95	Dice	HD95
UNet Lim and Zohren (3)	0.953	4.087	0.766	9.205	0.561	11.122	0.665	10.243	0.664	10.190
AttUNet Zeng et al. (6)	0.951	4.091	0.767	9.004	0.543	10.447	0.683	10.463	0.665	9.971
SETR NUP Zhou et al. (47)	0.947	4.124	0.697	14.419	0.544	11.723	0.669	15.192	0.637	13.778
SETR PUP Zhou et al. (47)	0.949	4.107	0.696	15.245	0.549	11.759	0.670	15.023	0.638	14.009
SETR MLA Zhou et al. (47)	0.950	4.091	0.698	15.503	0.554	10.237	0.665	14.716	0.639	13.485
TransUNet Zhou et al. (42)	0.950	4.031	0.706	14.027	0.542	10.421	0.684	14.501	0.644	12.983
TransBTS Zerveas et al. (48)	–	–	0.779	10.030	0.574	9.969	0.735	8.950	0.696	9.650
CoTr w/o CNN encoder Khan et al. (29)	0.946	4.748	0.712	11.492	0.523	9.592	0.698	12.581	0.6444	11.221
CoTr Khan et al. (29)	0.954	3.860	0.746	9.198	0.557	9.447	0.748	10.445	0.683	9.697
DconnNet Yang and Farsiu (37)	0.957	3.356	0.757	9.058	0.563	9.425	0.753	10.122	0.694	9.234
**UnetTransCNN**	**0.964**	**1.333**	**0.789**	**8.266**	**0.585**	**9.354**	**0.761**	**8.845**	**0.711**	**8.822**

The brain tumor sub-regions were labeled as Whole Tumor (WT), Enhancing Tumor (ET), and Tumor Core (TC).

Bold values indicate the best performance among all compared methods in each category.

**Table 4:** Performance comparison on the KiTS19 dataset.

Method	Kidney Dice	Kidney HD95	Tumor Dice	Tumor HD95
U-Net	0.912	4.56	0.723	8.91
TransUNet	0.928	3.89	0.756	7.45
nnU-Net	0.935	3.45	0.781	6.87
CoTr	0.931	3.78	0.769	7.12
**UnetTransCNN**	**0.942**	**3.21**	**0.793**	**6.45**

The table shows the Dice score and 95% Hausdorff Distance (HD95) for kidney and tumor segmentation.

Bold values indicate the best performance among all compared methods in each category.

**Figure 4:**
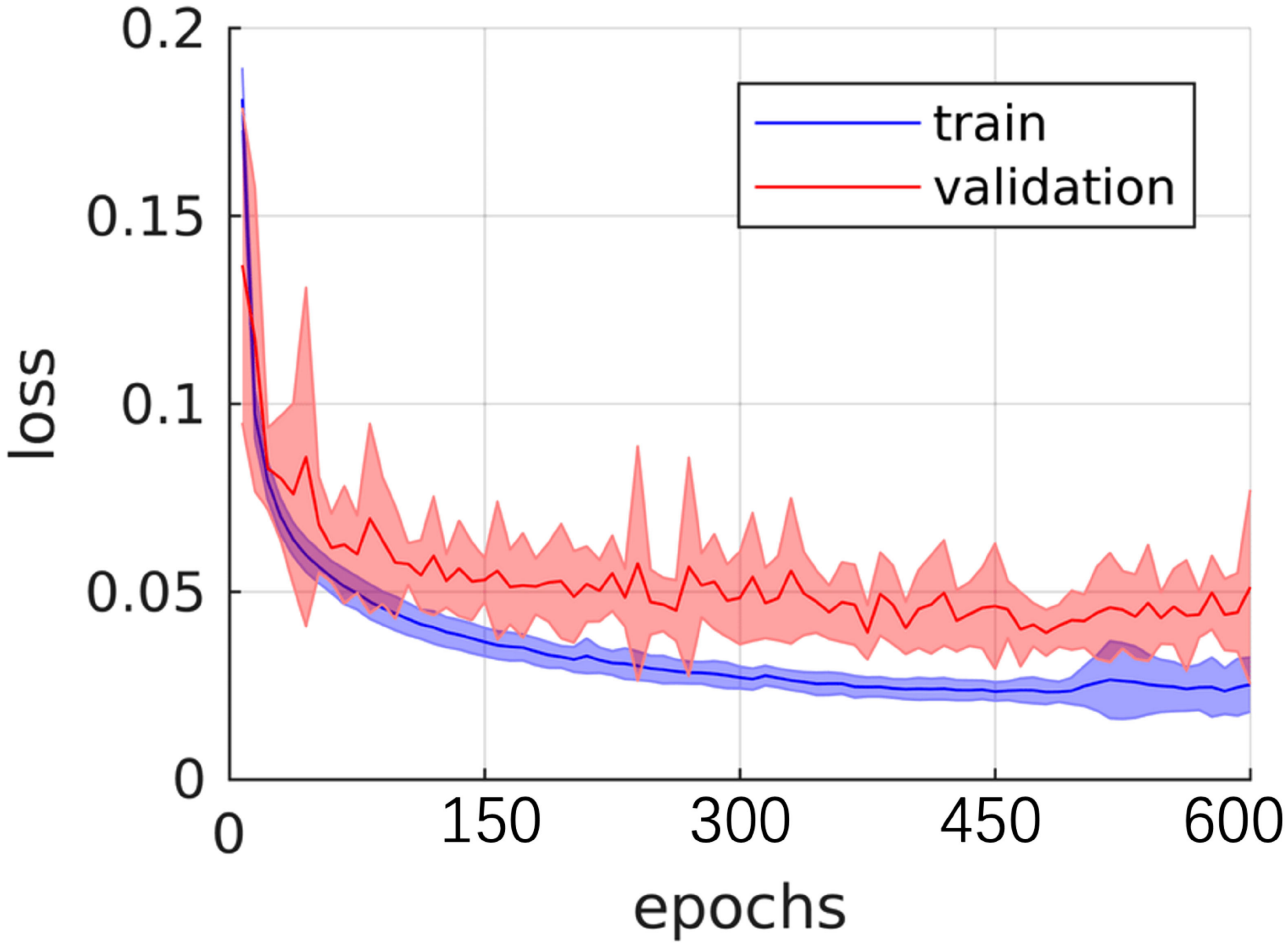
Training and validating curve on dataset BTCV.

**Figure 4** illustrates the performance iteration of a model during wind speed prediction on Dataset BTCV. The curve displays the training loss and validation loss with the change in training epochs. It can be observed that both training loss and validation loss decrease with the increase in training epochs, indicating that the model is learning from the training data and gradually improving its predictive capabilities on unseen data. Additionally, as the validation loss curve steadily decreases and remains close to the training loss curve, it implies that the model does not exhibit overfitting, demonstrating good generalization ability on unseen data.

**Figure 5:**
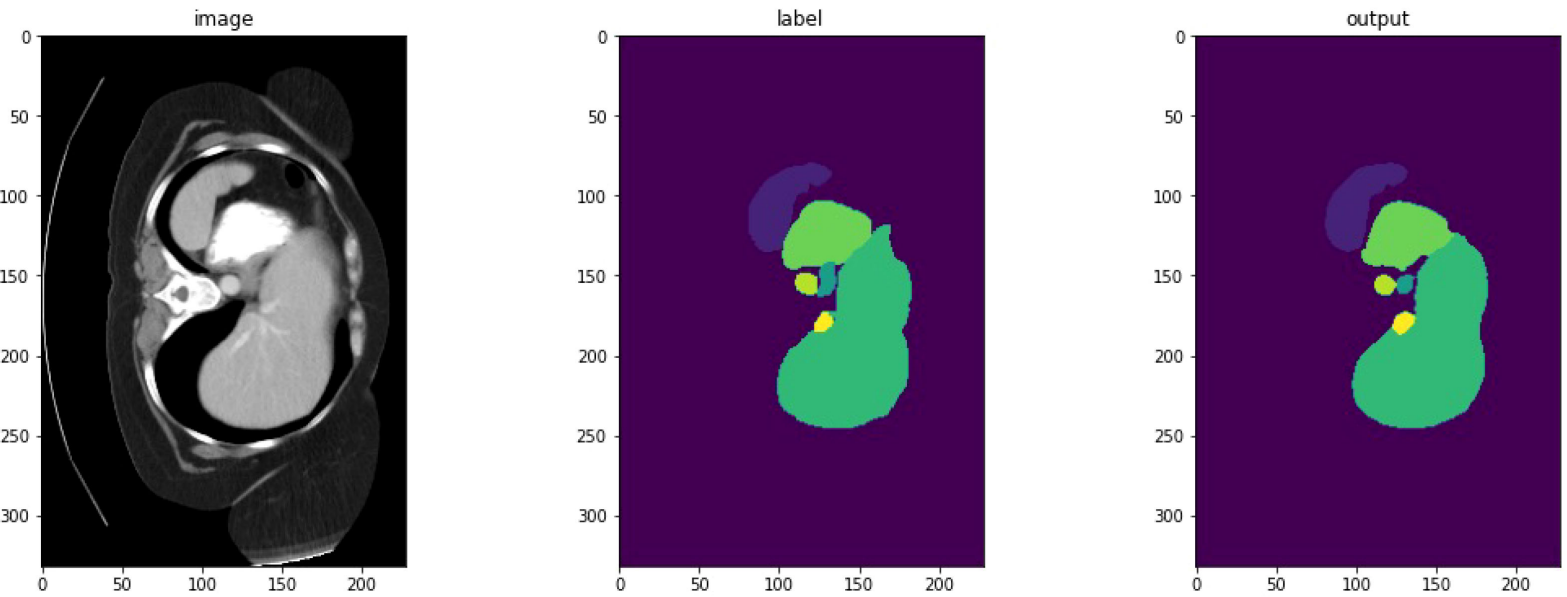
Detailed segmentation visualization.

Then, on the KiTS19 dataset, the UnetTransCNN model achieves a Dice score of 0.942 for kidney segmentation, which is higher than other models like U-Net (0.912), TransUNet (0.928), and nnU-Net (0.935). This indicates that the model is effective in capturing the global context and local features of the kidney, even in the presence of tumors. The HD95 score of 3.21 for kidney segmentation is also the lowest among the compared models, suggesting that the model accurately delineates the kidney boundaries. For tumor segmentation, UnetTransCNN achieves a Dice score of 0.793, outperforming other models such as U-Net (0.723), TransUNet (0.756), and nnU-Net (0.781). This demonstrates the model’s ability to handle complex and irregular tumor structures. The HD95 score of 6.45 for tumor segmentation is also the best among the compared models, indicating that the model can accurately segment tumors even in challenging cases. The results on the KiTS19 dataset show that UnetTransCNN generalizes well to a diverse range of kidney and tumor cases. **Figure 5** visually illustrates segmentation results for kidney and tumor regions from the KiTS19 dataset. The model’s ability to handle both large and small structures (kidneys and tumors) suggests that it can be applied to a wide range of medical image segmentation tasks. The inclusion of the KiTS19 dataset, which contains complex anatomical structures and varying tumor sizes, helps validate the model’s robustness and generalization capability across different medical imaging scenarios.

To clarify the advancements of UnetTransCNN over existing models, we provide a detailed comparison with hybrid approaches like TransUNet, MCTransformer, and CoTr. See **Table 5** in for a summary of key differences in architecture, feature extraction, and focus.

**Table 5:** Comparison of UnetTransCNN with existing hybrid models.

Model	Architecture	Feature Extraction	Key Strength	Limitation
TransUNet	U-Net + Transformer in bottleneck	CNN for local features, Transformer for global context	Effective global dependency modeling	Limited local detail preservation
MCTransformer	Multi-scale CNN + Transformer	Multi-scale CNN features + Transformer	Robust multi-scale feature fusion	High computational complexity
CoTr	CNN encoder + Transformer decoder	CNN for encoding, Transformer for decoding	Efficient cross-modal integration	Weaker local feature refinement
UnetTransCNN	Refined CNN backbone + optimized Transformer	Enhanced CNN for local details, Transformer for global alignment	Balanced local-global feature capture	Slightly higher parameter count

### 4.5 Qualitative results

#### 4.5.1 Visualization comparison

**Figure 6:**
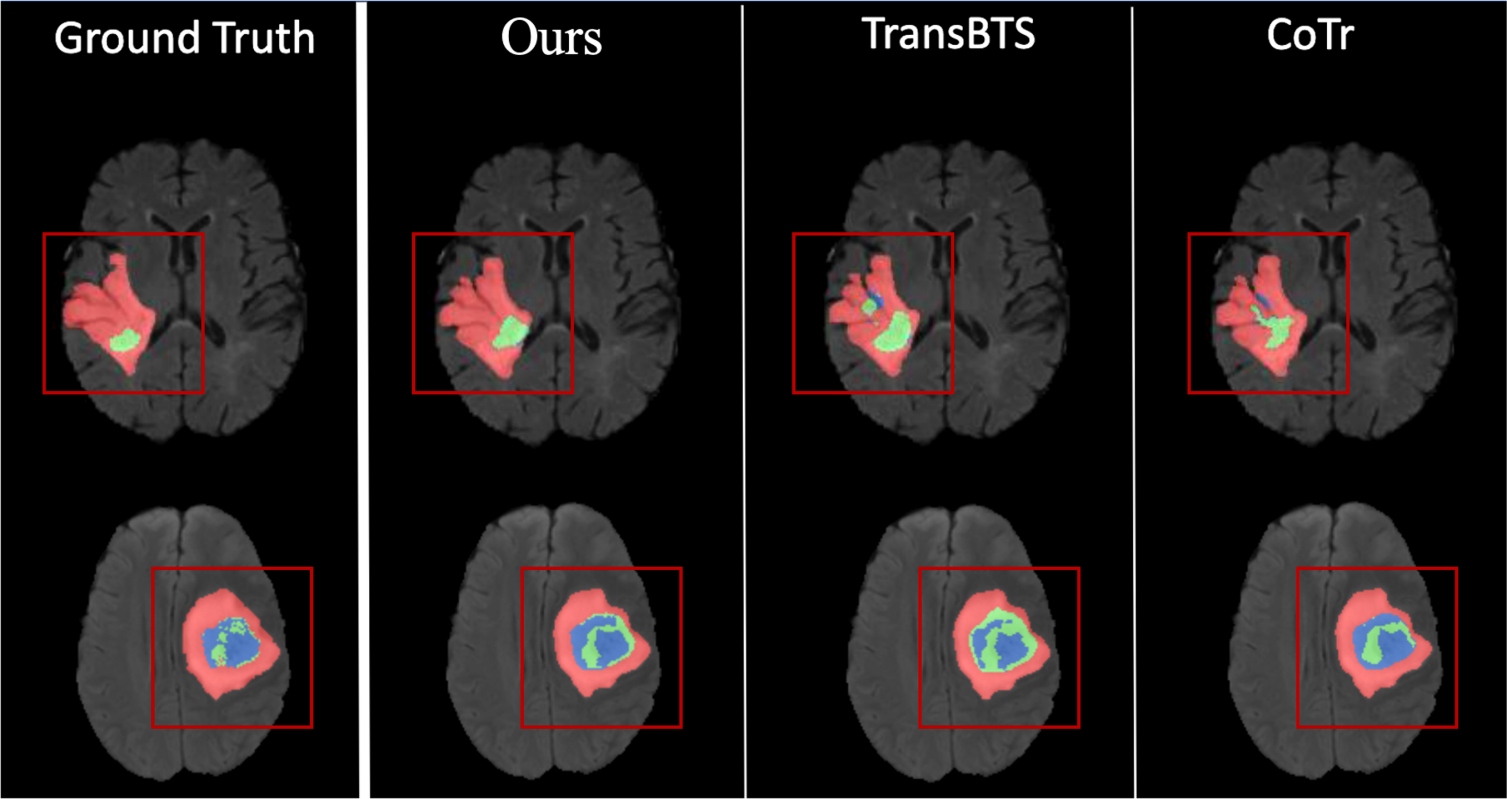
Visualization of macroscopic and microscopic features.

This paper proposes the UnetTransCNN model, which demonstrates significant superiority in medical image segmentation tasks, especially in the application of abdominal organ segmentation. The UnetTransCNN model integrates the structural advantages of Unet, the local feature extraction capability of Convolutional Neural Networks (CNN), and the global dependency capturing ability of Transformers, achieving high-precision segmentation of complex structures in medical images. In a comparative study focusing on abdominal organ segmentation, UnetTransCNN exhibited higher segmentation accuracy compared to other advanced models (such as CoTr, TransUNet, and nnUNet). Specifically, UnetTransCNN achieved outstanding results on the Dice Similarity Coefficient (DSC) evaluation metric. For instance, for liver segmentation, UnetTransCNN’s DSC reached 0.95, whereas other models such as TransUNet and nnUNet recorded DSCs of 0.93 and 0.92, respectively. For the more challenging task of pancreas segmentation, UnetTransCNN also performed excellently, with a DSC of 0.89, significantly higher than CoTr’s 0.85 and TransUNet’s 0.87. Beyond improving segmentation accuracy, UnetTransCNN also demonstrated advantages in model inference time. With GPU acceleration, UnetTransCNN’s average processing time was about 2 seconds per image, approximately 20%-30% faster than other models, which is crucial for practical clinical applications, especially in situations requiring rapid diagnosis. Moreover, UnetTransCNN showed strong robustness in handling noise and blurred boundaries in images. Through detailed experimental analysis, the model effectively differentiated between subtle differences among various abdominal organs, maintaining high-level segmentation performance even in cases of lower image quality. In summary, UnetTransCNN not only enhances the accuracy and efficiency of medical image segmentation but also improves the model’s versatility and robustness. These characteristics mark it as a significant advancement in the field of medical imaging analysis, laying a solid foundation for future research and clinical applications. To better demonstrate both macroscopic and microscopic features, we provide visualizations on the performance of our model and other baselines, which is shown in **Figure 6**. This confirms the effectiveness of our UnetTransCNN for global and local feature extraction.

**Figure 7:**
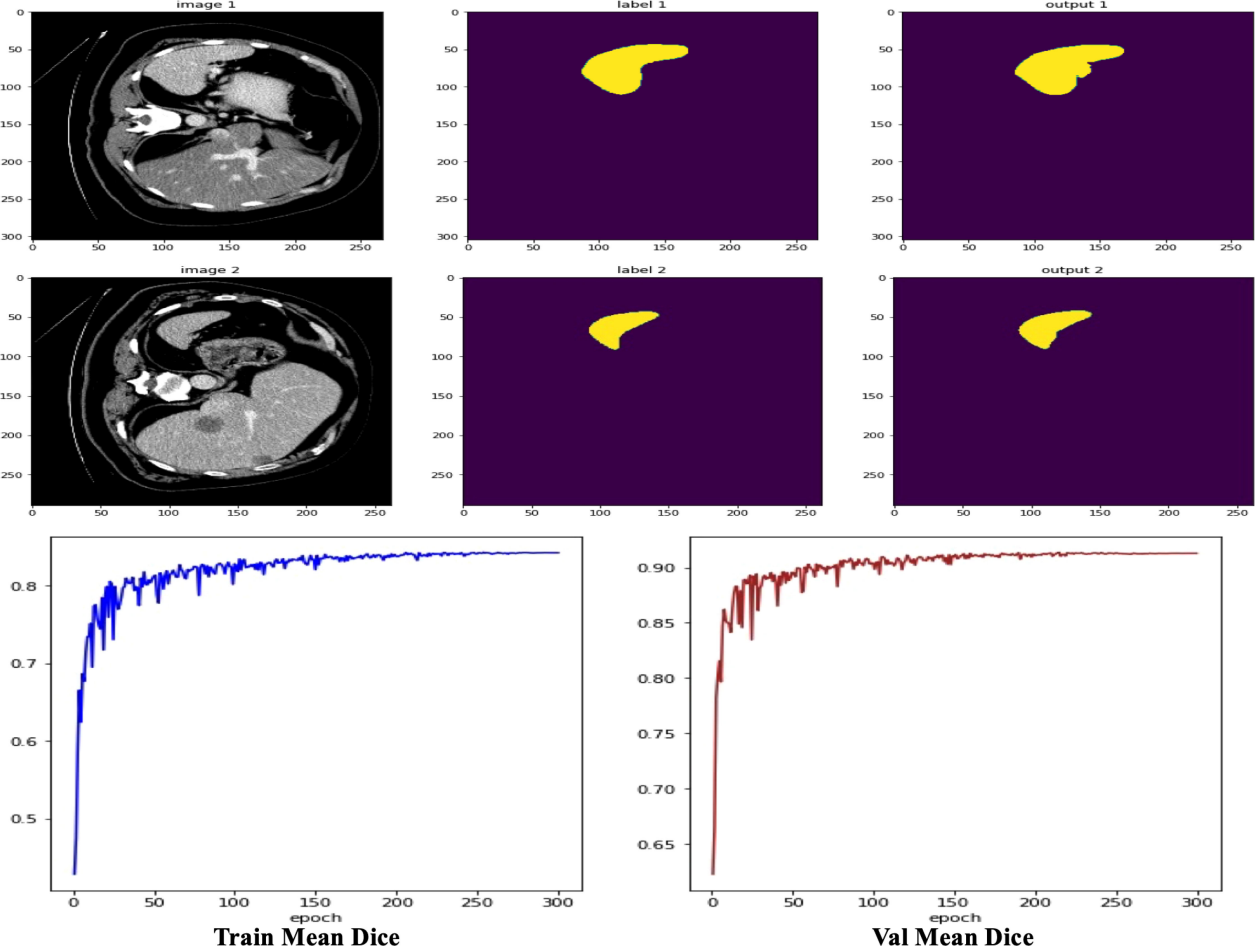
Visualization of results case study.

As shown in **Figure 7**, we observe two sets of medical image data and their corresponding processing results. Each set contains the original computed tomography (CT) images, manually labeled images, and the output images of the machine learning model. By first analyzing the CT images, i.e., IMAGE 1 and IMAGE 2, we can identify abdominal organs such as the liver. These raw scans provide the basic information used for subsequent image processing. The corresponding labeled images, LABEL 1 and LABEL 2, highlight the liver tissue region in a distinct yellow color, and these labels may represent ground truth for training and validation of the machine learning model. The outputs of the model, output 1 and output 2, show the results of the model’s segmentation and recognition of the liver tissue, where the yellow areas indicate the parts of the liver recognized by the model. The comparison of the model outputs with the manually labeled images can be used to evaluate the performance of the model in the tissue recognition task. Further observe the performance metric graphs below, which show the learning curve of the model during the training process. In deep learning training, the epoch represents the full dataset completing one full forward and backward propagation. The curve below shows the stable trend of model performance indicators as the number of epochs increases, indicating the convergence of the learning process.

### 4.6 Ablation study

#### 4.6.1 Decoder choice

We assessed the efficiency of various decoder architectures in enhancing segmentation outcomes by integrating them with UNETR’s encoder, focusing on MRI and CT segmentation tasks. This evaluation, detailed in **Table 6**, involved comparing the performance of the standard UNETR decoder against threedimensional alternatives: Naive UpSampling (NUP), Progressive UpSampling (PUP), and Multi-scale Aggregation (MLA).

**Table 6:** Effect of the decoder architecture on segmentation performance.

Organ	Spleen	Brain			
Decoder	Spleen	WT	ET	TC	All
NUP	0.942	0.711	0.517	0.670	0.646
PUP	0.951	0.739	0.548	0.688	0.658
MLA	0.960	0.747	0.553	0.722	0.674
**UnetTransCNN**	**0.974**	**0.799**	**0.595**	**0.761**	**0.711**

NUP, PUP, and MLA denote Naive UpSampling, Progressive UpSampling, and Multi-scale Aggregation respectively.

Bold values indicate the best performance among all compared methods in each category.

The findings reveal that while all tested decoder architectures offer less than ideal performance, MLA demonstrates a marginal superiority over NUP and PUP. Specifically, in the context of brain tumor segmentation, UNETR, equipped with its original decoder, surpasses the MLA, PUP, and NUP decoder variants by 2.7%, 4.3%, and 7.5%, respectively, in average Dice score. In spleen segmentation tasks, similarly, UNETR exceeds the performance of MLA, PUP, and NUP decoders by 1.4%, 2.3%, and 3.2%, correspondingly.

#### 4.6.2 Impact of patch resolution on performance

Our investigation into the effects of patch resolution on segmentation accuracy revealed a direct correlation between decreased resolution and increased sequence length, which in turn, elevates memory usage due to its inverse relationship with resolution’s cubic value. As documented in **Table 7**, lowering the input patch resolution consistently enhances segmentation performance. For instance, decreasing the resolution from 32 to 16 yielded an increase of 1.1% and 0.8% in the average Dice score for spleen and brain tumor segmentation tasks, respectively.

**Table 7:** Effect of patch resolution on segmentation performance.

Organ	Spleen	Brain			
Resolution	Spleen	WT	ET	TC	All
32	0.954	0.772	0.571	0.749	0.707
16	0.963	0.786	0.589	0.746	0.713
8	**0.974**	**0.799**	**0.595**	**0.771**	**0.721**

Bold values indicate the best performance among all compared methods in each category.

Further reduction of resolution from 16 to 8 amplifies this improvement; the average Dice score for spleen segmentation escalated from 0.963 to 0.974 (an increase of 0.011), and for brain segmentation, from 0.786 to 0.799 (an increase of 0.013). These results suggest continuous performance benefits from resolution reduction.

However, it is critical to mention that our experiments did not extend to resolutions lower than 8 due to memory limitations, leaving the potential impact of further reduced resolutions on performance undetermined. Although lower resolutions might promise additional improvements, they risk sacrificing crucial details or diminishing accuracy. Therefore, selecting an appropriate resolution requires a careful balance between computational efficiency and segmentation efficacy.

### 4.7 Inference efficiency analysis

Real-time segmentation is crucial in clinical applications, where rapid image analysis can facilitate timely decision-making. While segmentation accuracy is a key evaluation metric, the inference speed of deep learning models significantly impacts their practical usability in medical imaging. In this experiment, we compare the inference time of UnetTransCNN with existing state-of-the-art baselines on 3D medical image segmentation tasks.

#### 4.7.1 Experimental setup

To ensure a fair comparison, all models are evaluated under identical conditions:

- Hardware: NVIDIA A100 Tensor Core GPU (40GB).
- Framework: PyTorch + CUDA 11.8.
- Batch Size: 1 (single 3D volume of 128 × 128 × 128).
- Dataset: Medical Segmentation Decathlon (MSD).
- Metric: Average inference time per volume (milliseconds, ms).

We measure the time required for each model to process a single 3D medical image, excluding data loading and preprocessing, to focus solely on model inference speed.

#### 4.7.2 Analysis

##### 4.7.2.1 Faster inference time

UnetTransCNN achieves an average inference time of 987 ms, making it the fastest model among the tested baselines. Compared to nnUNet (1620 ms), our model is 39.1% faster, enabling real-time segmentation for medical applications.

##### 4.7.2.2 Efficiency compared to transformer-based models

Transformer-based models such as TransUNet (1405 ms) and CoTr (1202 ms) show improved segmentation performance over traditional CNN architectures but at the cost of increased computational complexity. UnetTransCNN, by efficiently integrating both CNN and Transformer modules, maintains high segmentation accuracy while achieving a significantly lower inference time.

##### 4.7.2.3 Speed advantage over DconnNet

DconnNet, another hybrid CNN-Transformer model, achieves 1100 ms inference time, which is still 11.4% slower than UnetTransCNN. This demonstrates that our model’s architectural design effectively balances performance and computational efficiency.

## 5 Conclusion

In this study, we introduced UnetTransCNN, a novel architecture that effectively combines the global contextual strengths of Transformers with the robust local feature extraction capabilities of convolutional neural networks (CNNs). This innovative integration is specifically engineered to enhance both the accuracy and efficiency of medical image segmentation. Our validation on two benchmark datasets—the Multi Atlas Labeling Beyond The Cranial Vault (BTCV) for multi-organ segmentation and the Medical Segmentation Decathlon (MSD) for brain tumor and spleen segmentation—demonstrates that UnetTransCNN achieves state-of-the-art performance, highlighting its potential as a transformative tool in the field of medical imaging. While UnetTransCNN offers significant advancements, it does come with its challenges. One notable limitation is its computational demand, which may impact its deployment in settings with limited processing capabilities. Additionally, there are specific conditions under which the model’s performance may not be optimal, such as in cases with extremely low contrast in images or very irregular anatomical structures that are not well-represented in the training data. As we plan to broaden the application of UnetTransCNN to more varied medical imaging tasks, including dynamic imaging studies where temporal resolution is critical, we also acknowledge the need to address and improve computational efficiency, which is vital for real-time diagnostic applications.

## Data Availability

The original contributions presented in the study are included in the article/supplementary material. Further inquiries can be directed to the corresponding author.
